# Multifunctional PVCL nanogels with redox-responsiveness enable enhanced MR imaging and ultrasound-promoted tumor chemotherapy

**DOI:** 10.7150/thno.43402

**Published:** 2020-03-15

**Authors:** Fang Xu, Jianzhi Zhu, Lizhou Lin, Changchang Zhang, Wenjie Sun, Yu Fan, Fangfang Yin, Jan C. M. van Hest, Han Wang, Lianfang Du, Xiangyang Shi

**Affiliations:** 1 State Key Laboratory for Modification of Chemical Fibers and Polymer Materials, International Joint Laboratory for Advanced Fiber and Low-dimension Materials, College of Chemistry, Chemical Engineering and Biotechnology, Donghua University, Shanghai 201620, People's Republic of China.; 2 Department of Ultrasound, Shanghai General Hospital, Shanghai Jiao Tong University School of Medicine, Shanghai 200080, People's Republic of China.; 3 Bio-Organic Chemistry, Institute for Complex Molecular Systems, Eindhoven University of Technology, 5600 MB Eindhoven, the Netherlands.; 4 Department of Radiology, Shanghai General Hospital, Shanghai Jiao Tong University School of Medicine, Shanghai 200080, People's Republic of China.

**Keywords:** nanogels, manganese dioxide nanoparticles, ultrasound-targeted microbubble destruction, magnetic resonance imaging, chemotherapy

## Abstract

Development of versatile nanoplatforms that simultaneously integrate therapeutic and diagnostic features for stimuli-responsive delivery to tumors remains a great challenge. In this work, we report a novel intelligent redox-responsive hybrid nanosystem composed of MnO_2_ nanoparticles (NPs) and doxorubicin (DOX) co-loaded within poly(N-vinylcaprolactam) nanogels (PVCL NGs) for magnetic resonance (MR) imaging-guided and ultrasound-targeted microbubble destruction (UTMD)-promoted tumor chemotherapy.

**Methods**: PVCL NGs were first synthesized *via* a precipitation polymerization method, decorated with amines using ethylenediamine, and loaded with MnO_2_ NPs through oxidation with permanganate and DOX *via* physical encapsulation and Mn-N coordination bonding. The as-prepared DOX/MnO_2_@PVCL NGs were well characterized. UTMD-promoted cellular uptake and therapeutic efficacy of the hybrid NGs were assessed *in vitro*, and a xenografted tumor model was used to test the NGs for MR imaging and UTMD-promoted tumor therapy *in vivo.*

**Results**: The as-prepared DOX/MnO_2_@PVCL NGs with a size of 106.8 nm display excellent colloidal stability, favorable biocompatibility, and redox-responsiveness to the reductive intracellular environment and tumor tissues having a relatively high glutathione (GSH) concentration that can trigger the synchronous release of Mn^2+^ for enhanced T_1_-weighted MR imaging and DOX for enhanced cancer chemotherapy. Moreover, the DOX/MnO_2_@PVCL NGs upon the UTMD-promotion exhibit a significantly enhanced tumor growth inhibition effect toward subcutaneous B16 melanoma owing to the UTMD-improved cellular internalization and tumor penetration.

**Conclusion**: Our work thereby proposes a promising theranostic nanoplatform for stimuli-responsive T_1_-weighted MR imaging-guided tumor chemotherapy.

## Introduction

Constructing intelligent stimuli-responsive multifunctional nanosystems that simultaneously incorporate diagnostic-imaging performance and therapeutic function is of great importance but still remains a challenge in the nanomedicine field 1-7. Recently, manganese dioxide (MnO_2_) nanoparticles (NPs) have been demonstrated as a promising diagnostic and therapeutic nanoplatform due to their tumor-specific imaging performance, high cargo-loading capacity and glutathione (GSH)-triggered drug release properties 8-13. In particular, MnO_2_ NPs have been demonstrated as an alternative contrast agent for T_1_-weighted magnetic resonance (MR) imaging as a potential substitute of the clinical gadolinium (Gd)-based contrast agents owing to their superior biocompatibility and ability to be reduced to form Mn(II) ions 14. However, the biomedical applications of MnO_2_ NPs are limited by their poor stability, especially under the physiological conditions. Therefore, various strategies such as employing bovine serum albumin (BSA) as stabilizer 15,16 and surface PEGylation modification 14 have been investigated to address this issue. Nevertheless, the introduction of BSA and PEGylation potentially suffers from immunogenicity and PEGylation-induced decrease of cellular uptake 17-19, thereby restricting their further clinical applications. Consequently, pioneering novel strategies to establish MnO_2_-based nanocarriers with intelligent performance is desired in the blossoming field of nanomedicine.

Nanogels (NGs) have recently been attracted considerable attention in the field of nanomedicine 20-24. This is mainly due to the fact that NGs exhibit excellent hydrophilicity, fluidity and deformability, and the employment of NGs as nanoplatforms could contribute to the improved stability, prolonged circulation time, desirable biocompatibility and enhanced cellular uptake 25-27. In our earlier research, we systematically investigated the fabrication, characterization and utilization of poly(N-vinylcaprolactam) (PVCL) NGs for different biomedical applications 28,29. Notably, Gd-loaded PVCL NGs with desirable physicochemical property and good biocompatibility were exploited as an effective contrast agent for enhanced T_1_-weighted MR imaging of subcutaneous tumor model 28. In addition, PVCL NGs with a stimuli-responsive feature have been investigated as a drug carrier for cancer therapy 23,30. Based on these previous efforts, the integration of PVCL NGs with MnO_2_ nanoplatforms may provide a promising approach to realize the biomedical applications of MnO_2_ NPs for cancer theranostics.

The effective accumulation of therapeutic agents within the solid tumor region is one of the most challenging obstacles 31,32. The ultrasound-targeted microbubble destruction (UTMD) technology offers an encouraging tool to boost the targeted delivery of nanosystems in the tumor site 33,34. In general, with the assistance of UTMD, the tumor capillary permeability and the clathrin-based endocytosis along the irradiation route are promoted by the UTMD-produced sonoporation, thus increasing the accumulation and cellular internalization of nanoplatforms in the tumor site 35. In our previous work, we illustrated that the introduction of UTMD technology could significantly enhance the co-delivery of gemcitabine and microRNA inhibitor through a dendrimer-based platform for effective pancreatic cancer treatments 33. Therefore, the utilization of UTMD holds a great potential to boost the accumulation of MnO_2_ nanocarriers in the specific tumor region, hence improving the diagnostic sensitivity and therapeutic outcome.

**Figure 1:**
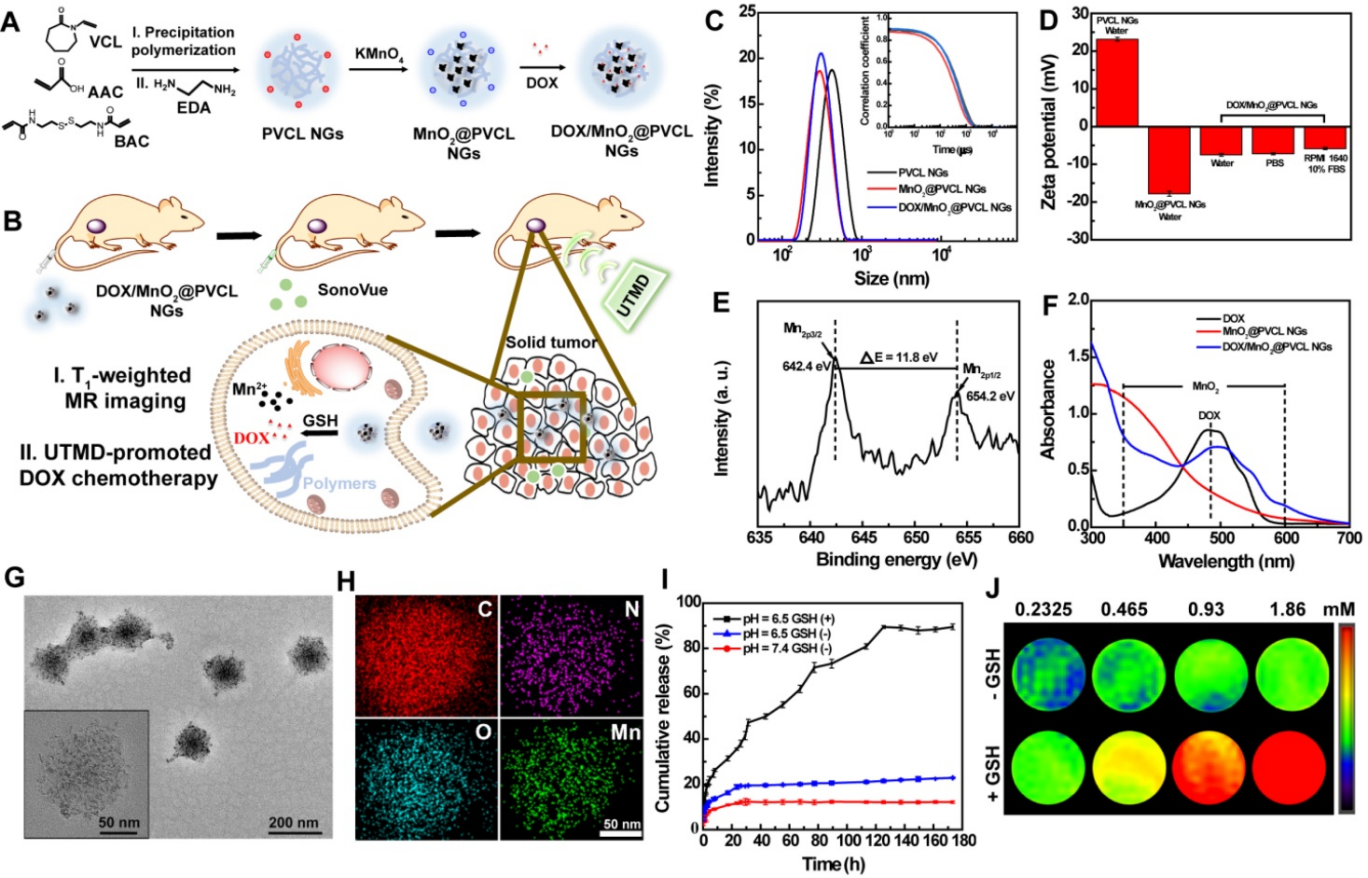
** (A)** Synthetic route for the fabrication of DOX/MnO_2_@PVCL NGs. **(B)** Schematic illustration of the utilization of DOX/MnO_2_@PVCL NGs for UTMD-promoted delivery of DOX/MnO_2_@PVCL NGs for MR imaging-guided cancer chemotherapy. **(C)** Hydrodynamic size distribution and relative correlation coefficient (inset) of PVCL, MnO_2_@PVCL and DOX/MnO_2_@PVCL NGs in water. **(D)** Zeta potentials of PVCL, MnO_2_@PVCL and DOX/MnO_2_@PVCL NGs in different aqueous media (n = 3). **(E)** XPS spectrum of DOX/MnO_2_@PVCL NGs. **(F)** UV-vis spectra of free DOX, MnO_2_@PVCL and DOX/MnO_2_@PVCL NGs. **(G)** TEM image and **(H)** EDX elemental mapping analysis of DOX/MnO_2_@PVCL NGs. **(I)** DOX release profile from DOX/MnO_2_@PVCL NGs at pH 7.4/6.5 in the presence or absence of GSH (10 mM). Data are shown as mean ± SD (n = 3). **(J)** Pseudo-colored T_1_-weighted MR images of DOX/MnO_2_@PVCL NGs with different Mn concentrations in the presence or absence of GSH (10 mM). The color bar from blue to red indicates the gradual increase of MR signal intensity.

Herein, we for the first time describe the UTMD-promoted delivery of MnO_2_ NPs and doxorubicin (DOX) through a PVCL NG system for T_1_-weighted MR imaging-guided cancer chemotherapy (Figure 1A-B). To construct this nanosystem, PVCL NGs were synthesized in the presence of a crosslinker of N, N'-bis(acryloyl)cystamine (BAC) containing disulfide bond *via* a precipitation polymerization method, aminated to introduce primary amine groups on their surface and within their interior, loaded with MnO_2_ NPs *via* a redox reaction between the NG amine groups and permanganate, and encapsulated with DOX, a typical broad-spectrum chemotherapeutic drug, through physical interaction and Mn-N coordinate bonds. The as-prepared DOX/MnO_2_@PVCL NGs were systematically characterized through different techniques. The redox-responsive T_1_ MR relaxometry of MnO_2_ NPs and the release kinetics of DOX in the presence of GSH were investigated* in vitro*, and the UTMD-promoted cellular uptake and therapeutic efficacy of the hybrid NGs were assessed *in vitro*. Finally, we explored the use of the DOX/MnO_2_@PVCL NGs for MR imaging of a xenografted tumor model and the UTMD-promoted tumor therapy *in vivo.* To our knowledge, this is the very first example related to the development of PVCL-based hybrid NG system for imaging and tumor chemotherapy.

## Results and Discussion

### Synthesis and characterization of DOX/MnO_2_@PVCL NGs

The main aim of this study is to design redox-responsive DOX/MnO_2_@PVCL NGs for enhanced tumor MR imaging and anticancer drug delivery in the GSH-rich tumor microenvironment. Specifically, GSH-mediated NG dissolution would lead to the breakup and disintegration of the DOX/MnO_2_@PVCL NGs to release Mn^2+^ for markedly enhanced T_1_-weighted MR imaging, and simultaneously to fast release DOX at the tumor region for enhancing chemotherapy. Notably, upon the promotion of UTMD, the DOX/MnO_2_@PVCL NGs would exhibit a significantly enhanced tumor growth inhibition effect owing to the improved cellular internalization and tumor penetration of the NGs.

The route for the fabrication of DOX/MnO_2_@PVCL NGs is shown in Figure 1A. Typically, PVCL NGs were first formed in aqueous solution *via* a precipitation polymerization method 28, 29 using N-vinylcaprolactam (VCL) and acrylic acid (AAc) as co-monomers and GSH-cleavable BAC as a crosslinker, followed by amination with excessive ethylenediamine (EDA) to introduce amine groups on their surface and within their interior. Through a redox reaction between the NG amine groups and permanganate 36, MnO_2_ NPs were loaded within the PVCL NGs. Different mass feed ratios of the PVCL NGs/permanganate (1:0.1, 1:0.25, 1:0.5, 1:0.75 or 1:1) were employed for the optimization. Finally, DOX was encapsulated through physical interaction and Mn-N coordinate bonds 37 to obtain the DOX/MnO_2_@PVCL NGs.

Dynamic light scattering (DLS) and zeta-potential measurements were first employed to characterize the as-prepared NGs. As shown in Figure 1C-D, the PVCL NGs exhibited a hydrodynamic size of 390.7 ± 3.11 nm and positive surface potential of 23.1 ± 0.53 mV due to the EDA-mediated amination. After the formation of MnO_2_ NPs, the overall hydrodynamic size of the NGs is smaller than the pristine PVCL NGs (Table S1). Interestingly, with the increase of the feed amount of KMnO_4_, the hydrodynamic diameter of the MnO_2_@PVCL NGs, compared to that of PVCL NGs, first decreased possibly due to the compression of the structural softness by the solid MnO_2_, whereafter slightly increased because of the following MnO_2_ growth. Therefore, the feed ratio of PVCL NGs to KMnO_4_ was set at 1:0.5 to render the MnO_2_@PVCL NGs with the smallest hydrodynamic size of 258.1 ± 2.75 nm. It is also interesting to note that the MnO_2_@PVCL NGs exhibit a negative surface potential of -17.2 ± 0.63 mV, likely attributed to the oxidation of the NG amine groups by KMnO_4_ and the introduction of negatively charged MnO_2_ 14,38. This further suggests the successful loading of MnO_2_. Further encapsulation of DOX led to the formation of the hybrid DOX/MnO_2_@PVCL NGs with a hydrodynamic size of 278.1 ± 2.75 nm and a slightly negative surface charge of -7.51 ± 0.37 mV.

The size and stability of the DOX/MnO_2_@PVCL NGs in phosphate-buffered saline (PBS) and cell culture medium were assessed by DLS (Figures S1 and S2), demonstrating their narrow size distribution and relatively long-term stability, which is amenable for their biomedical applications. UV-vis spectra and X-ray photoelectron spectroscopy (XPS) were then employed to further verify the formation of DOX/MnO_2_@PVCL NGs. As can be seen from Figure 1E, two peaks centered at 642.4 and 654.2 eV in the XPS spectrum could be assigned to the Mn_2p3/2_ and Mn_2p1/2_ spin-orbit peaks of MnO_2_, respectively, evidencing the successful formation of MnO_2_. The characteristic UV-vis absorption features appearing at 350-600 nm and 480 nm, respectively imply the successful co-loading of colloidal MnO_2_ NPs and DOX within the NGs (Figure 1F).

Scanning electron microscopy (SEM) and transmission electron microscopy (TEM) were used to observe the morphology and size of pristine PVCL, MnO_2_@PVCL and DOX/MnO_2_@PVCL NGs (Figures S3, S4 and S5). PVCL NGs, DOX-free and DOX-loaded MnO_2_@PVCL NGs display a spherical morphology with a narrow size distribution, and have an average size of 109.1, 80.1 and 106.8 nm, respectively. Interestingly, the hydrodynamic size of the MnO_2_@PVCL NGs measured by DLS is dramatically larger than those measured by SEM and TEM. This could be attributed to the fact that DLS measures the hydrodynamic size of the hybrid NGs that are swollen with a surface hydration layer, while SEM or TEM only measures the size of the dried and shrunken NGs. TEM image reveals that the MnO_2_ NPs were homogeneously distributed within the DOX/MnO_2_@PVCL NGs (Figure 1G) with a mean particle size of 3.9 nm (Figure S6). The uniform distribution of MnO_2_ NPs within the NGs can be further validated by energy-dispersive X-ray (EDX) elemental mapping analysis (Figure 1H), where the elements of C, N, O, and Mn are well distributed within each NG entirely. The loading percentage of MnO_2_ NPs within the NGs was measured to be 18.54% by inductively coupled plasma-optical emission spectrometry (ICP-OES). Meanwhile, through UV-vis spectral analysis, the DOX loading efficiency and loading content within the NGs were determined to be 81.22% and 33.34%, respectively. The much higher DOX loading capacity of the MnO_2_@PVCL NGs than that of similar MnO_2_-free PVCL NGs 22,30 could be attributed to the fact that the pre-loaded MnO_2_ NPs may strongly absorb DOX through Mn-N coordination bonding, in consistence with the literature 8,14.

### Redox-responsive DOX release from the hybrid NGs

The redox-triggered DOX release behavior of the developed DOX/MnO_2_@PVCL NGs was next investigated. As can be seen in Figure 1I, the NGs showed only around 10% DOX release within 7 days under physiological pH of 7.4 in the absence of GSH, indicating their favorable stability to avoid the leakage of loaded drug during *in vivo* circulation process. Under the mild acidic tumor microenvironment pH of 6.5 in the absence of GSH, the released DOX content slightly increased to around 20% at the same time point, presumably due to the increased water solubility of the DOX drug under slightly acidic pH condition, and also the partial decomposition of MnO_2_ NPs by the H^+^ to form Mn(II) ions to facilitate the release of DOX adsorbed onto the surface of MnO_2_ NPs 14. Importantly, under pH 6.5 in the presence of GSH (10 mM), the hybrid NGs could quickly release DOX within 2 h to have a DOX release percentage of 17.0%, and at 7 days the DOX release could reach 89.5%. This may be due to the fact that the NGs were subjected to internal gel network break, namely the GSH-mediated break of S-S bond within the NGs to lead to swollen and ruptured internal gel network. This can be confirmed by the significantly increased hydrodynamic size (4427 nm) of NGs in water containing 10 mM of GSH (Figure S7).

### MR relaxometry

In general, Mn^2+^ with five unpaired 3d electrons can be employed as an effective T_1_-weighted MR imaging contrast agent. We next explored the MR imaging performance of the NGs. As shown in Figure 1J, no obvious T_1_ MR enhancement was observed in the absence of GSH, whereas the NGs displayed a concentration-dependent MR contrast enhancement after they were treated with 10 mM of GSH for 1 h. Notably, the r_1_ relaxivity of the NGs in the presence of GSH was determined to be 8.33 mM^-1^s^-1^, much higher than that of NGs in the absence of GSH (0.04 mM^-1^s^-1^, Figure S8), and also 1.8 folds higher than that of the clinical Magnevist® (4.56 mM^-1^s^-1^ under 0.5 Tesla) 28. This indicates that redox-responsive NGs may function as a positive contrast agent for T_1_-weighted MR imaging of tumors having a high local concentration of GSH.

### *In vitro* cytotoxicity and cellular uptake assays

**Figure 2:**
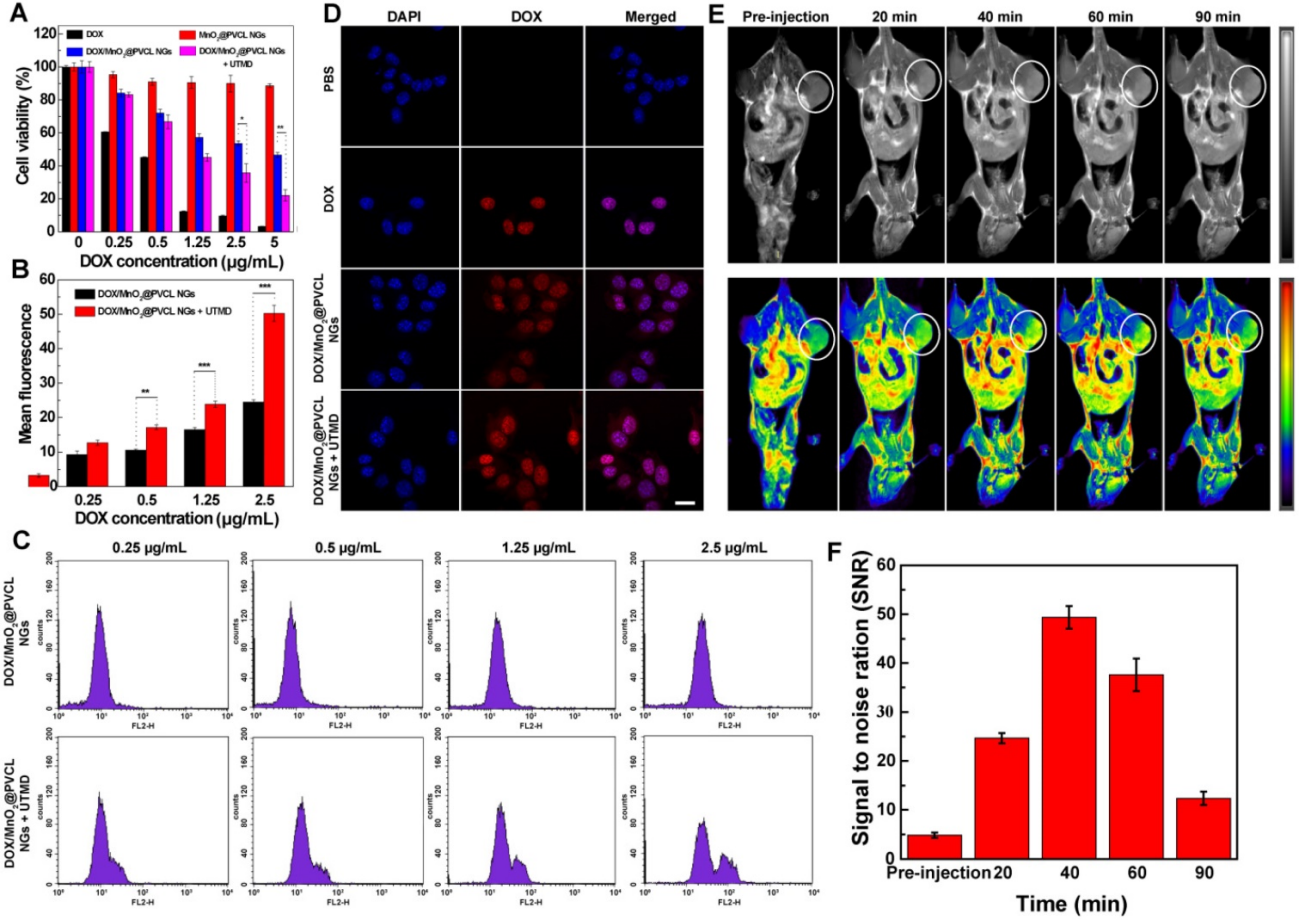
** (A)** Cell viability of B16 cells treated with free DOX, MnO_2_@PVCL, DOX/MnO_2_@PVCL and DOX/MnO_2_@PVCL NGs + UTMD at different DOX concentrations for 24 h using CCK-8 assays (n = 5). **(B)** Quantified FACS analysis of DOX mean fluorescence intensity and **(C)** FACS histograms of B16 cells treated with DOX/MnO_2_@PVCL NGs in the presence and absence of UTMD at different DOX concentrations (n = 3). **(D)** CLSM images of B16 cells incubated with free DOX, DOX/MnO_2_@PVCL and DOX/MnO_2_@PVCL NGs + UTMD. Scale bar: 20 µm for each panel. **(E)** *In vivo* T_1_-weighted MR images (grey and pseudo-color) of mice bearing subcutaneous B16 tumors before and at different time points post-injection of DOX/MnO_2_@PVCL NGs ([DOX] = 5 mg/kg, in 200 µL of PBS for each mouse). The color bar from blue to red indicates the gradual increase of MR signal intensity. **(F)** MR signal to noise ratio (SNR) of the tumor region at different time points post-injection of DOX/MnO_2_@PVCL NGs (n = 3).

The toxicity of DOX/MnO_2_@PVCL NGs towards B16 melanoma cancer cells was evaluated using a standard cell counting kit-8 (CCK-8) assay in the absence and presence of UTMD treatment 33. Figure 2A shows that the viability of B16 cells incubated with MnO_2_@PVCL NGs after 24 h does not show obvious change at the given concentrations when compared to the control cells treated with PBS. This implies that the drug-free MnO_2_@PVCL NGs possess a good cytocompatibility. To be reasonable, all groups of DOX-loaded NGs exhibit lower cytotoxicity than free DOX, likely due to the sustained DOX release *in vitro*. Notably, the employment of UTMD treatment significantly improves the therapeutic efficacy of DOX-loaded NGs especially at DOX concentration above 2.5 µg/mL (p < 0.05), despite that single UTMD treatment has no obvious influence on the in vitro cell viability as proven in our previous work 33. This may be attributed to the UTMD-enhanced cellular uptake of NGs 33,39.

Flow cytometry (FACS) and confocal laser scanning microscopy (CLSM) were next employed to explore the *in vitro* cellular uptake behavior of DOX/MnO_2_@PVCL NGs in the presence or absence of UTMD treatment. FACS data (Figure 2B-C) reveal that the fluorescence intensity of B16 cells is much higher after UTMD treatment than without UTMD treatment, especially at DOX concentration > 0.5 µg/mL (p < 0.01). This enhanced cellular uptake was further verified by confocal microscopic imaging of B16 cells. We can see from Figure 2D that the DOX fluorescence merged well with the cell nuclei, suggesting that DOX could be released from the NGs and enter the cell nuclei. It is worthwhile to note that B16 cells treated with UTMD display higher DOX fluorescence intensity than those without UTMD treatment. Overall, flow cytometry and CLSM studies demonstrate that B16 cells could take up more NGs under UTMD treatment to induce enhanced cancer cell killing than without UTMD.

### *In vivo* T_1_-weighted MR imaging of subcutaneous B16 tumor model

To test the MR imaging performance of DOX/MnO_2_@PVCL NGs, a mouse model of subcutaneous B16 tumors was built up. As elucidated in Figure 2E, the tumor region displays a relatively low MR signal before injection and starts to be brightened at 20 min post-injection. The MR contrast enhancement was the highest at a peak time of 40 min post-injection, and then declines with the time post-injection possibly due to the elimination of Mn^2+^. Further quantitative evaluation of the MR signal to noise ratio (SNR) of tumors reveals the same trend (Figure 2F). Therefore, *in vivo* imaging results demonstrate that the developed DOX/MnO_2_@PVCL NGs can be employed as a positive contrast agent for T_1_-weighted MR imaging of tumors.

### UTMD-promoted antitumor effect* in vivo*

**Figure 3:**
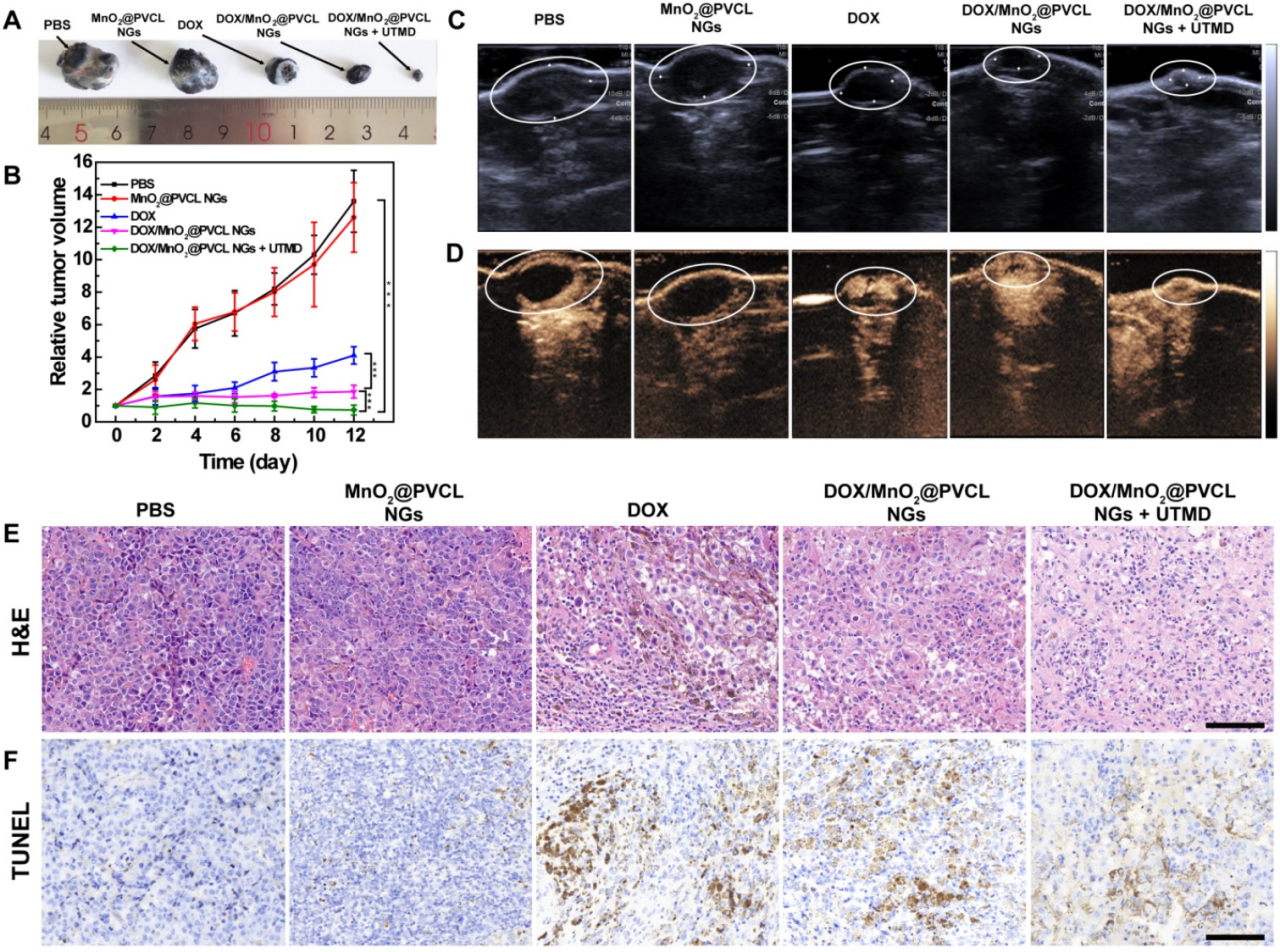
** (A)** Photograph of tumors on day 12 after different treatments. **(B)** Tumor growth curves after different treatments (n = 5). Tumor volumes (V) were normalized to their initial values (V_0_). **(C)** B-mode US images and **(D)** CEUS images of the tumor on day 12 after different treatments. **(E)** H&E staining and **(F)** TUNEL staining of tumor slices taken on day 12 after different treatments. Scale bar for (E) and (F) represents 200 µm for each panel.

After demonstrating the effective accumulation of the developed NGs within the tumor site for T_1_-weighted MR imaging, we hereby investigated the UTMD-promoted tumor chemotherapy effect. Mice bearing B16 tumors with a size of around 100 mm^3^ were randomly divided into 5 groups, which were treated respectively with (1) PBS, (2) MnO_2_@PVCL NGs, (3) free DOX, (4) DOX/MnO_2_@PVCL NGs and (5) DOX/MnO_2_@PVCL NGs + UTMD at the corresponding DOX dose of 5 mg/kg. The UTMD treatment was performed right following the injection of NGs referring to our previous work 33, 34. Notably, we have demonstrated that single UTMD treatment has no obvious inhibition effect on the tumor growth 33, here we did not include the single UTMD treatment group. The therapeutic effect was evaluated by monitoring the tumor size changes, and the representative tumor image of each group was recorded after the therapy (Figure 3A-B). The mice in the PBS and MnO_2_@PVCL NGs groups displayed a similar tumor growth rate, indicating that the MnO_2_@PVCL NGs possess good biocompatibility and have no therapeutic effect. Interestingly, both the DOX/MnO_2_@PVCL NGs and DOX could significantly suppress the tumor growth, but the DOX/MnO_2_@PVCL NGs displayed an enhanced inhibition effect compared to free DOX. This is possibly due to enhanced permeability and retention (EPR)-based passive targeting of the NGs to the tumor site. Under UTMD treatment, the DOX/MnO_2_@PVCL NGs exhibit the most effective tumor suppression effect over all other groups, likely due to the enhanced NG accumulation and penetration within the enlarged pores of tumor tissue.

B-mode ultrasound (US) imaging was next employed to visualize the tumor size after the therapy process. It is clear in Figure 3C that tumor-bearing mice treated with DOX/MnO_2_@PVCL NGs + UTMD displayed a significantly decreased tumor volume when compared to the other groups, verifying the effective antitumor ability and the enhancing role of UTMD during the therapy process. Since the intratumoral blood flow could be used as an indicator for prognosis 40, contrast-enhanced ultrasound (CEUS) imaging was performed to reveal the tumor vascular condition. As shown in Figure 3D, the tumor treated with DOX/MnO_2_@PVCL NGs + UTMD displays the most abundant intratumoral blood perfusion, suggesting that the DOX/MnO_2_@PVCL NGs + UTMD treatment displays superior satisfactory prognosis over other groups. This was also confirmed by quantitative analysis of echo intensity of the tumor region in different groups (Figure S9). The hematoxylin-eosin (H&E) and TdT-mediated dUTP Nick-End Labeling (TUNEL) staining of the tumor after different treatments reveal that combined treatment of DOX/MnO_2_@PVCL NGs + UTMD shows the highest level of tumor cell necrosis and apoptosis among all groups (Figure 3E-F). Quantitative analysis of TUNEL images (Figure S10) shows that the apoptosis rate of tumor cells in the group of DOX/MnO_2_@PVCL NGs + UTMD (68.4%) is significantly higher than those of PBS (1.8%), MnO_2_@PVCL NGs (7.9%), DOX (37.8%) and DOX/MnO_2_@PVCL NGs (52.5%) groups. The *in vivo* therapy results suggest that the DOX/MnO_2_@PVCL NGs with UTMD-promotion are able to significantly improve the therapeutic efficacy towards subcutaneous B16 melanoma.

### *In vivo* biodistribution and biocompatibility evaluation

**Figure 4:**
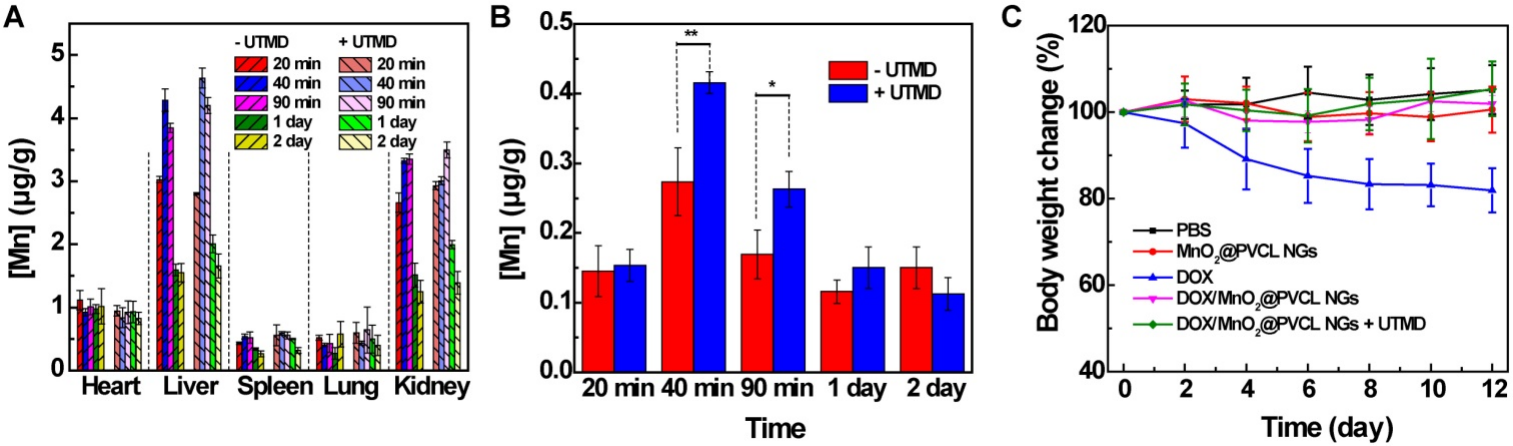
*In vivo* biodistribution of Mn in the **(A)** major organs of the mice (including the heart, liver, spleen, lung, and kidney) and **(B)** tumor at different time points post single intravenous injection of DOX/MnO_2_@PVCL NGs in the absence and presence of UTMD ([DOX] = 5 mg/kg, in 200 µL of PBS for each mouse, n = 3). **(C)** Mouse body weight changes in different groups over the *in vivo* therapy process (n = 5).

The *in vivo* metabolic behavior of the DOX/MnO_2_@PVCL NGs after intravenous injection was investigated by quantifying the biodistribution of Mn element in different organs including the heart, liver, spleen, lung and kidney and tumor at different time points post-injection using ICP-OES. We can see from Figure 4A that the developed NGs are basically metabolized *via* liver and kidney. Notably, only a small part of NGs was distributed within the spleen and lung, indicating that the NGs possessed a good size distribution and no obvious particle aggregation occurred during the circulation 41. It seems that the metabolization process of the hybrid NGs in the presence of UTMD is similar to that in the absence of UTMD, suggesting that the introduction of UTMD treatment does not alter the *in vivo* biodistribution behaviour of the NGs. However, in the presence of UTMD, the Mn uptake in the tumors can be promoted, especially at the earlier time points of 40 and 90 min, the tumor Mn uptake is much higher after UTMD treatments than without UTMD (Figure 4B).

In order to estimate the *in vivo* toxicity of the developed NGs, the body weight change of tumor-bearing mice during the therapy process was recorded for all groups (Figure 4C). The free DOX administration leads to a significant body weight decrease, whereas no obvious body weight changes occur in other groups, demonstrating that the employment of MnO_2_@PVCL NGs as nanocarriers could avoid the side effect resulting from free-DOX treatment. To further evaluate the biocompatibility of the hybrid NGs, the major organs including heart, liver, spleen, lung, and kidney of the mice after *in vivo* therapy process were harvested for H&E staining. As can be seen in Figure S11, for free DOX group, acute necrosis, interstitial edema and tubular dilatation were observed in renal tissue; liver tissues exhibited necrosis, inflammatory infiltration, and even fibrosis; the lung showed enlarged pulmonary alveoli with inflammatory infiltration; and the spleen showed typical necrotic cells. However, no significant pathological abnormalities such as inflammatory infiltrate, morphological changes and necrosis for all organs were found in the mice treated with DOX/MnO_2_@PVCL NGs. Further, to validate the biosafety of the hybrid NGs, we performed *in vitro* hemolysis assay and blood routine test *in vivo*. At the studied NG concentrations, the red blood cells (RBC) display a hemolysis percentage less than the threshold value of 5% 42, implying their favorable hemocompatibility (Figure S12). Additionally, blood routine tests show that the injection of the DOX/MnO_2_@PVCL NGs for 7 and 14 days does not seem to impact the routine biochemical parameters when compared to the PBS control group (Figure S13). Therefore, it is reasonable to conclude that the DOX/MnO_2_@PVCL NGs exert their therapeutic effect *in vivo* against B16 melanoma without any significant systemic toxicity.

## Conclusions

In summary, we developed a facile approach to creating intelligent redox-responsive DOX/MnO_2_@PVCL NGs for MR imaging-guided and UTMD-promoted cancer chemotherapy. Through a simple *in-situ* redox reaction to oxidize the primary amine groups of the NGs, MnO_2_ NPs can be loaded within the PVCL NGs crosslinked by disulfide bond for subsequent loading of anticancer drug DOX. The developed NGs with a size of 106.8 nm display excellent stability, satisfactory biocompatibility, and redox-responsiveness to tumor tissue having a high local concentration of GSH, thus allowing for synchronous release of Mn^2+^ as positive contrast agents for T_1_-weighted MR imaging and DOX for cancer chemotherapy. Upon the promotion of UTMD, the DOX/MnO_2_@PVCL NGs exhibit enhanced therapeutic efficacy owing to the improved tumor penetration and tumor cell internalization. The designed novel theranostic hybrid NGs thereby hold a great promise for theranostics of different cancer types.

## Experiment Section

### Preparation of PVCL NGs

PVCL NGs with carboxylic groups were first synthesized using a precipitation polymerization approach referring to the literature 28,29. Primary amine groups were then introduced to the above NGs *via* an EDC/NHS-mediated coupling reaction between the carboxyl groups of the above NGs and the amine groups of EDA to obtain the aminated PVCL NGs. Briefly, EDC (287.55 mg) and NHS (172.635 mg) in 6 mL of water were added to the above NG dispersion (210 mg, in 30 mL water) under stirring for 2 h to activate the carboxyl groups. Excess EDA (200.4 μL) was quickly injected to the above solution and the reaction was kept at room temperature for 3 days. Subsequently, the mixture was dialyzed against water for 3 days to remove the impurity. The purified NGs solution was stored at 4 °C and a small portion was freeze-dried to determine the mass concentration.

### Synthesis of MnO_2_@PVCL NGs

MnO_2_ NPs were loaded within the PVCL NGs via a redox reaction between the primary amine groups of the PVCL NGs and potassium permanganate 36. Briefly, KMnO_4_ solution (5 mg/mL) with different volumes was added to 10 mL of PVCL NGs dispersion (7 mg/mL) using a syringe pump with a flow rate of 0.1 mL/min. The mass ratio of PVCL NGs to KMnO_4_ was set as 1: 0.1, 1: 0.25, 1: 0.5, 1: 0.75, and 1: 1, respectively in order to optimize the preparation of the NGs. The mixture was stirred overnight, and purified through dialysis against water for 3 days. A fraction of purified brown liquid was lyophilized to determine the mass concentration and the left was stored at 4 °C for further use.

### Synthesis of DOX/MnO_2_@PVCL NGs

DOX was encapsulated within the MnO_2_@PVCL NGs by physical interaction and Mn-N coordinate bonds. Briefly, an aqueous DOX solution (1.54 mg/mL, 3.25 mL in water) was added into a solution of MnO_2_@PVCL NGs (5.88 mg/mL, 1.7 mL in water), followed by adjusting the solution pH to 8 with NaOH (1 M). The mixture was stirred at room temperature in the dark for 24 h. The dispersion was then centrifuged (13 000 rpm, 30 min) to collect the precipitate (the final DOX/MnO_2_@PVCL NGs), and the supernatant containing non-loaded free DOX was also collected for quantification of the DOX loading percentage and efficiency. The DOX loading efficiency and loading content were determined by UV-vis spectroscopic analysis of DOX (λ = 480 nm) from the initial DOX solution and the supernatant DOX solution after the encapsulation process with the following formulas: Loading efficiency (%) = [(weight of loaded DOX)/(initial weight of DOX)] × 100%; and loading content (%) = [(weight of loaded DOX)/(total weight of DOX/MnO_2_@PVCL NGs)] × 100%. Animal experiments were performed according to the guidelines of the Institutional Animal Care and Use Committees (IACUC) of Donghua University and the policy of the National Ministry of Health. See full experimental details in Supporting Information.

## Supplementary Material

Supplementary experimental section, figures, and table.
