# Supplementary material for: Multifunctional PVCL nanogels with redox-responsiveness enable enhanced MR imaging and ultrasound-promoted tumor chemotherapy
Source: Theranostics. 2020 Mar 15;10(10):4349–58. doi: 10.7150/thno.43402 (PMC7150492; doi:10.7150/thno.43402)
Supplement: Supplementary file 1 — Supplementary experimental section, figures, and table. [file thnov10p4349s1.pdf]

Supporting Information

Multifunctional PVCL nanogels with redox-responsiveness enable enhanced MR imaging and ultrasound-promoted tumor chemotherapy

Fang Xu[a1], Jianzhi Zhu[a,c1], Lizhou Lin[b1], Changchang Zhang[a], Wenjie Sun[a], Yu Fan[a], Fangfang Yin[d], Jan C. M. van Hest[c], Han Wang[d], Lianfang Du[b]* and Xiangyang Shi[a]*

[a] State Key Laboratory for Modification of Chemical Fibers and Polymer Materials, International Joint Laboratory for Advanced Fiber and Low-dimension Materials, College of Chemistry, Chemical Engineering and Biotechnology, Donghua University, Shanghai 201620, People's Republic of China

[b] Department of Ultrasound, Shanghai General Hospital, Shanghai Jiao Tong University School of Medicine, Shanghai 200080, People's Republic of China

[c] Bio-Organic Chemistry, Institute for Complex Molecular Systems, Eindhoven University of Technology, 5600 MB Eindhoven, the Netherlands

[d] Department of Radiology, Shanghai General Hospital, Shanghai Jiao Tong University School of Medicine, Shanghai 200080, People's Republic of China

Keywords: nanogels; manganese dioxide nanoparticles; ultrasound-targeted microbubble destruction; magnetic resonance imaging; chemotherapy

---

* Corresponding author. E-mail: xshi@dhu.edu.cn (X. Shi) and dulf_sh@163.com (L. Du)

[1] Authors contributed equally to this work.

Experiment Section

Materials

N-vinylcaprolactam (VCL), ethylenediamine (EDA), potassium permanganate and glutathione (GSH) were obtained from J&K Scientific (Shanghai, China). Acrylic acid (AAc) (99%) was purchased from Aladdin (Shanghai, China). Sodium dodecyl sulfate (SDS) was from Sigma-Aldrich (St. Louis, MO). N, N′-bis(acryloyl)cystamine (BAC) was from Alfa Aesar (Shanghai, China). 2, 2-Azobis [N-(2-carboxyethyl)-2-methylpropionamidine] (ACMA) was from Wako Pure Chemical Industries (Osaka, Japan). Doxorubicin hydrochloride (DOX·HCl) was from Beijing Huangfeng Pharmaceutical Co., Ltd. (Beijing, China). 1-Ethyl-3-(3-dimethylaminopropyl) carbodiimide hydrochloride (EDC) and N-hydroxysuccinimide (NHS) were from GL Biochem (Shanghai, China). RPMI 1640 Medium and fetal bovine serum (FBS) were from Gibco (Carlsbad, CA). Penicillin and streptomycin (P/S) were from Gino Biomedical Technology Co., Ltd. (Hangzhou, China). Cell counting kit-8 (CCK-8) was acquired from 7sea Biotech. Co., Ltd. (Shanghai, China). Cellulose dialysis membranes with a molecular weight cut-off (MWCO) at 12,000-14,000 were acquired from Shanghai Yuanye Biotechnology Corporation (Shanghai, China). Water used in all experiments with a typical resistivity higher than 18.2 MΩ.cm was purified by a Milli-Q Plus 185 water purification system (Boston, MA).

Preparation of PVCL NGs

PVCL NGs with carboxylic groups were first synthesized using a precipitation polymerization approach referring to the literature [1, 2]. In brief, VCL (1.878 g), BAC (98.28 mg) and SDS (20 mg) were co-dissolved in 120 mL of water under stirring at 70 ^{o}C for 30 min with N_2 protection to obtain a homogenous solution. Then, ACMA (70 mg, in 5 mL water) was added to the above solution to initiate the polymerization. After 5 to 8 min, AAc (108 mg, in 25 mL water) was dropped into the above mixture. The sequential addition of chemicals was under N_2 protection, and the whole reaction was maintained at 70 ^{o}C for 4 h under N_2 protection. Afterwards, the reaction mixture was cooled

down to room temperature (RT) and the obtained white dispersion was dialyzed against water with a dialysis membrane having an MWCO of 12,000-14,000 for 3 days to remove the unreacted monomers. A fraction of the dialysis liquid was subjected to lyophilization to determine the mass concentration and the left was stored at 4 ^{o}C for further use.

Primary amine groups were then introduced to the above NGs *via* an EDC/NHS-mediated coupling reaction between the carboxyl groups of the above NGs and the amine groups of EDA to obtain the aminated PVCL NGs. Briefly, EDC (287.55 mg) and NHS (172.635 mg) in 6 mL of water were added to the above NG dispersion (210 mg, in 30 mL water) under stirring for 2 h to activate the carboxyl groups. Excess EDA (200.4 μL) was quickly injected to the above solution and the reaction was kept at RT for 3 days. Subsequently, the mixture was dialyzed against water using protocols as described above for 3 days to remove the impurity. The purified NGs solution was stored at 4 ^{o}C and a small portion was freeze-dried to determine the mass concentration.

Synthesis of MnO_2@PVCL NGs

MnO_2 NPs were loaded within the PVCL NGs *via* a redox reaction between the primary amine groups of the PVCL NGs and potassium permanganate [3]. Briefly, different volume of $KMnO_4$ solution (5 mg/mL) was added to 10 mL of PVCL NGs dispersion (7 mg/mL) using a syringe pump with a flow rate of 0.1 mL/min. The mass ratio of PVCL NGs to $KMnO_4$ was set as 1 : 0.1, 1 : 0.25, 1 : 0.5, 1 : 0.75, and 1 : 1, respectively in order to optimize the preparation of the NGs. The mixture was stirred overnight, purified through dialysis against water for 3 days. A fraction of purified brown liquid was lyophilized to determine the mass concentration and the left was stored at 4 ^{o}C for further use.

Synthesis of DOX/MnO_2@PVCL NGs

DOX was encapsulated within the MnO_2@PVCL NGs by physical interaction and Mn-N coordinate bonds. Briefly, an aqueous DOX solution (1.54 mg/mL, 3.25 mL in water) was added into a solution of MnO_2@PVCL NGs (5.88 mg/mL, 1.7 mL in water), followed by adjusting the

solution pH to 8 with NaOH (1 M). The mixture was stirred at RT in the dark for 24 h. The dispersion was then centrifuged (13 000 rpm, 30 min) to collect the precipitate (the final DOX/MnO_2@PVCL NGs), and the supernatant containing non-loaded free DOX was also collected for quantification of the DOX loading percentage and efficiency. The DOX loading efficiency and loading content were determined by UV-vis spectroscopic analysis of DOX (λ = 480 nm) from the initial DOX solution and the supernatant DOX solution after the encapsulation process with the following formulas: Loading efficiency (%) = [(weight of loaded DOX)/(initial weight of DOX)] × 100%; and loading content (%) = [(weight of loaded DOX)/(total weight of DOX/MnO_2@PVCL NGs)] × 100%.

Characterization Techniques

Dynamic light scattering (DLS) and zeta potential measurements were carried out using a Malvern Zetasizer Nano ZS model ZEN3600 (Worcestershire, UK) equipped with a standard 633 nm laser. The morphology of the MnO_2@PVCL NGs was observed using scanning electron microscope (SEM, S-4800 analytical electron microscope, Tokyo, Japan) at a voltage of 15 kV. The sample was prepared by dropping an NG suspension (1 mg/mL, 5 μL) onto aluminum foil, followed by air drying and sputter coating of a gold film with a thickness of 10 nm. Transmission electron microscopy (TEM) was performed using a JEOL 2010F electron microscope (Tokyo, Japan) at an operating voltage of 200 kV. One drop of the DOX/MnO_2@PVCL NGs in water (1 mg/mL) was deposited onto a carbon-coated copper grid and air-dried before measurements. The particle size distribution was measured using Image J 1.40 G software (http://rsb.info.nih.gov/ij/download.html). For each sample, at least 200 MnO_2@PVCL NGs, DOX/MnO_2@PVCL NGs or MnO_2 NPs were randomly selected from SEM or TEM images. UV-vis spectroscopy was carried out using a Lambda 25 UV-vis spectrophotometer (PerkinElmer, Waltham, MA). X-ray photoelectron spectroscopy (XPS) data were obtained with an Escalab 250Xi spectrometer (ThermoFisher Scientific, Waltham, MA), equipped with an analyzer mode (pass energy of 50 eV) and an Al Kα X-ray source. Mn

concentration was determined with inductively coupled plasma-optical emission spectroscopy (ICP-OES, Leeman Prodigy, Hudson, NH). Confocal laser scanning microscope (CLSM) images were captured by ZEISS LSM 700 (Jena, Germany). Becton Dickinson FACScan flow cytometer (FACS, Franklin, CA) was used to determine the cellular uptake of the NGs.

Magnetic Resonance (MR) Relaxometry

DOX/MnO_2@PVCL NGs were diluted to different concentrations and incubated overnight in the presence or absence of GSH (10 mM). T_1 MR relaxometry was performed on a 0.5 T NMI20 Analyzing and Imaging system (Shanghai NIUMAG Corporation, Shanghai, China) with the following parameters: TR = 400 ms, TE = 20 ms, resolution = 156 mm × 156 mm, and section thickness = 0.5 mm. The r_1 relaxivity was determined through linear fitting of the inverse T_1 relaxation time ($1/T_1$) as a function of Mn concentration. The pseudo-colored T_1-weighted MR images of the above solutions were captured on a 3.0 T MR imaging system (Discovery MR750, GE Healthcare, Milwaukee, WI) acquired using a SE/2D sequence with the following parameters: TR = 400 ms, TE = 12.2 ms, NEX = 4.00, matrix = 256 × 256, slice thickness = 2 mm, slice space = 0.8 mm, and FOV = 12 cm.

DOX Release from Hybrid NGs

DOX/MnO_2@PVCL NGs (1 mg) dispersed in 1 mL of phosphate buffer (pH 7.4 or 6.5) in the presence or absence of GSH (10 mM) were placed in a dialysis bag (MWCO = 12,000 -14,000), and then submerged into 9 mL of the corresponding buffer. The system was kept at 37 ^{o}C under constant shaking. Then, 1 mL of the buffer medium was pipetted out at scheduled time intervals and the volume of the outer phase buffer medium was maintained constant by replenishing 1 mL of the corresponding buffer solution. The experiment was performed in triplicate for each sample. The DOX concentration was determined by the UV-vis spectrometry to quantify the DOX absorption at 480 nm. The GSH-triggered disintegration was further investigated by measuring the hydrodynamic size change of the NGs in the presence of GSH (10 mM).

Cell Culture

B16 melanoma cancer cells were regularly cultured and passaged in RPMI-1640 medium supplemented with 10% FBS and 1% P/S in a Thermo Scientific cell incubator (Waltham, MA) at 37 ^{o}C and 5% CO_2.

***In Vitro* Ultrasound-Targeted Microbubble Destruction (UTMD) Treatment of Cells**

UTMD treatment was conducted using a therapeutic ultrasound machine (PHYSIOMED ELEKTROMEDIZIN, Schnaittach, Germany). According to our previous work [4], the procedure was set as follows: B16 cells were suspended with medium containing NGs and SonoVue and subsequently exposed to the ultrasound apparatus with the following optimized parameters: 0.4 W/cm^2, 1 MHz, 20% microbubbles, PRF 1 kHz, and 30 s. The processed cell suspension was then seeded into 12-well or 6-well plate for 6 h to study the influence of UTMD on the cellular uptake behavior of NGs. Cells treated with phosphate buffered saline (PBS) were used as control. To investigate the UTMD-promoted therapeutic efficacy of the NGs, the seeded cells were treated with medium containing NGs and SonoVue, UTMD treated for 30 s, and further incubated for 24 h before regular cell viability assay (see below).

***In Vitro* Cytotoxicity Assay**

B16 cells were seeded in a 96-well plate at a density of 8×10^3 cells per well and cultured overnight. The next day, the cells were incubated with fresh medium containing free DOX, MnO_2@PVCL NGs or DOX/MnO_2@PVCL NGs at DOX concentrations of 0.25, 0.5, 1, 2.5 and 5 μg/mL, respectively and cultured for another 24 h. For DOX-free MnO_2@PVCL NGs, the NG concentrations corresponded to the DOX-containing NGs. After rinsing 3 times with PBS, the cells were incubated with 100 μL of culture medium without FBS but supplemented with 10% CCK-8 for 3 h. The absorbance at 450 nm was recorded on the microplate reader (Multiskan MK3, Thermo Scientific, Waltham, MA). For each sample, 5 parallel wells were analyzed to give a mean value and standard deviation. To study the UTMD-enhanced cytotoxicity, *in vitro* UTMD treatment was

involved as aforementioned. The cytotoxicity was then measured following the same procedure mentioned above.

***In Vitro* Cellular Uptake**

The cellular uptake behavior of DOX/MnO_2@PVCL NGs in the presence or absence of UTMD treatment was explored by FACS and CLSM. Briefly, B16 cells were seeded at a density of 2×10^5 cells/well and cultured overnight. Fresh medium containing DOX/MnO_2@PVCL NGs with different DOX concentrations of 0.25, 0.5, 1, and 2.5 μg/mL, respectively was then incubated with the cells for 6 h. The culture medium was then removed and the cells were washed with PBS for 3 times. The adherent B16 cells were digested, centrifuged, and redispersed in PBS (1 mL) before flow cytometry analysis (for cells cultured in 12-well plates) to determine the fluorescence intensity of cells, and observed by CLSM (for cells cultured in 6-well plate and incubated with NGs at a DOX concentration of 2.5 μg/mL). To verify the UTMD-enhanced cellular uptake, protocols were referred to the above.

T_1-Weighted MR Imaging of a Subcutaneous Tumor Model

Animal experiments were performed according to the guidelines of the Institutional Animal Care and Use Committees (IACUC) of Donghua University and the policy of the National Ministry of Health. ICR mice (15-20 g) were purchased from Shanghai Slac Laboratory Animal Center (Shanghai, China). To establish a subcutaneous tumor model, 100 μL of B16 cell suspension (2×10^6 cells/mL) was subcutaneously injected into the right back of each mouse. When the tumor volume reaches 100-200 mm^3, each mouse was tail-vein intravenously injected with DOX/MnO_2@PVCL NGs ([DOX] = 5 mg/kg, in 200 μL of PBS). T_1-weighted MR images were acquired at different time points (20, 40, 60 and 90 min, respectively) post-injection on a 3.0 T MR imaging system (Discovery MR750, GE Healthcare, Milwaukee, WI) equipped with a custom-built rodent receiver coil (Chenguang Med Tech, Shanghai, China) using the parameters of TE = 20 ms, TR =519 ms, FOV = 100 × 100 mm, and matrix=336 × 333. The mice were anesthetized by

intraperitoneal injection of pentobarbital sodium (40 mg/kg) and immobilized in the rodent receiver coil using medical adhesive tape during the imaging process.

In Vivo Antitumor Efficacy

Mice bearing B16 subcutaneous tumors were randomly divided into 5 groups (5 mice for each group) which were separately treated with (1) PBS, (2) free DOX, (3) MnO_2@PVCL NGs, (4) DOX/MnO_2@PVCL NGs and (5) DOX/MnO_2@PVCL NGs + UTMD, with the corresponding DOX dose of 5 mg/kg. The mice were injected with the related materials via the tail vein every 2 days for one week. For the DOX/MnO_2@PVCL NGs + UTMD group, SonoVue (1.18 mg/mL, in 0.2 mL saline) was intravenously injected right following the injection of NGs, and the UTMD was performed using a US transducer (PHYSIOSON-BASIC, PHYSIOMED ELEKTROMEDIZIN, Schnaittach, Germany) positioning above the tumor region (1 MHz, 0.4 W/cm^2, 20% microbubbles, and 2 min) [5, 6]. In the process of therapy, the tumor volume ($V = a \times b^2/2$, a represents the tumor length and b the tumor width) and mice body weight were recorded every other day for 12 days.

B-mode ultrasound (US) imaging was used to visualize the tumor size using a clinical diagnostic ultrasound scanner (LOGIQ E9, GE, Fairfield, CT). In addition, mice after the treatment course were injected with SonoVue (1.18 mg/mL, 0.1 mL saline), and contrast-enhanced ultrasound (CEUS) imaging was conducted using the same probe (LOGIQ E9, ML 6-15 MHz) for the contrast mode at a low mechanical index (0.12). All CEUS images were collected under the same conditions (20 mm depth and 5 gains). In the end, the mice were anesthetized and sacrificed. The tumor pathological changes were analyzed by hematoxylin-eosin (H&E) and TdT-mediated dUTP Nick-End Labeling staining. To assess the organ toxicity of NGs, the major organs including heart, liver, spleen, lung, and kidney were harvested for H&E staining.

In Vivo Biodistribution

To explore the *in vivo* biodistribution of NGs, mice bearing B16 tumors were treated with DOX/MnO_2@PVCL NGs at a DOX dosage of 5 mg/kg in the presence or absence of UTMD, and were euthanized at different time points post-injection (20 min, 40 min, 90 min, 1 day, and 2 days, respectively). The major organs including the heart, liver, spleen, lungs, kidneys and tumor were extracted, weighed, digested by nitrohydrochloric acid overnight and then analyzed by ICP-OES to quantify Mn element. The tumor-bearing mice injected with PBS were used as control.

Hemolysis Assay and Blood Routine Tests

Hemolysis assay and blood routine tests were performed to evaluate the biosafety of the hybrid NGs. The healthy mice were anaesthetized and the eyeballs were removed to collect blood samples. Hemolysis assay was performed according to the literature [7]. Briefly, 1.5 mL of blood was diluted with 3.5 mL of PBS, then the pure red blood cells (RBCs) were obtained *via* centrifugation/redispersion (2000 rpm, 10 min, 3 times). The obtained RBCs were diluted with 5 mL of PBS. To test the hemolysis effect, 0.1 mL of the above RBC suspension was respectively added to 0.9 mL of water (positive control), 0.9 mL of PBS (negative control) and 0.9 mL of PBS containing MnO_2@PVCL NGs with different concentrations (25-200 μg/mL). The mixtures were incubated at 37 °C for 2 h. Afterwards, all the samples were centrifuged at 13000 rpm for 20 min, and the absorbance of the supernatant at 541 nm was measured by UV-vis spectrometry. The hemolysis percentage of each sample was calculated according to the following formula: hemolysis rate (%) = (sample absorbance − negative control absorbance) / (positive control absorbance − negative control absorbance) × 100%.

For blood routine tests, healthy mice were intravenously injected with the DOX/MnO_2@PVCL NGs ([DOX] = 5 mg/kg, in 200 μL of PBS for each mouse) or PBS as control. Blood samples were obtained by removing the mice eyeball after the mice were sacrificed at 7 or 14 days post-injection

(n = 3 for each group) and analyzed by Mindray BC-2800vet Hematology Analyzer (Shenzhen Mindray Bio-Medical Electronics Co., Ltd., Shenzhen, China).

Statistical Analysis

One-way analysis of variance (ANOVA) method was used to evaluate the significance of the experimental data with a significance level (p-value) of 0.05. The data were marked with (*) for $p < 0.05$, (**) for $p < 0.01$, (***) for $p < 0.001$, respectively. All experimental data were displayed as the mean ± standard deviation ($n \geq 3$).

Table S1. Hydrodynamic size, polydisperse index and zeta potential of MnO_2@PVCL NGs with different PVCL NGs/$KMnO_4$ feeding mass ratios.

Feed mass ratio (PVCL NGs/$KMnO_4$)	Hydrodynamic size (nm)	Polydisperse index (PDI)	Zeta potential (mV)
1 : 0.1	360.0 ± 5.27	0.22 ± 0.03	-11.8 ± 0.53
1 : 0.25	325.3 ± 1.84	0.21 ± 0.04	-13.7 ± 0.76
1 : 0.5	258.1 ± 2.75	0.11 ± 0.02	-17.2 ± 0.63
1 : 0.75	275.3 ± 5.67	0.14 ± 0.01	-21.1 ± 0.28
1 : 1	293.4 ± 4.29	0.10 ± 0.02	-20.6 ± 0.68

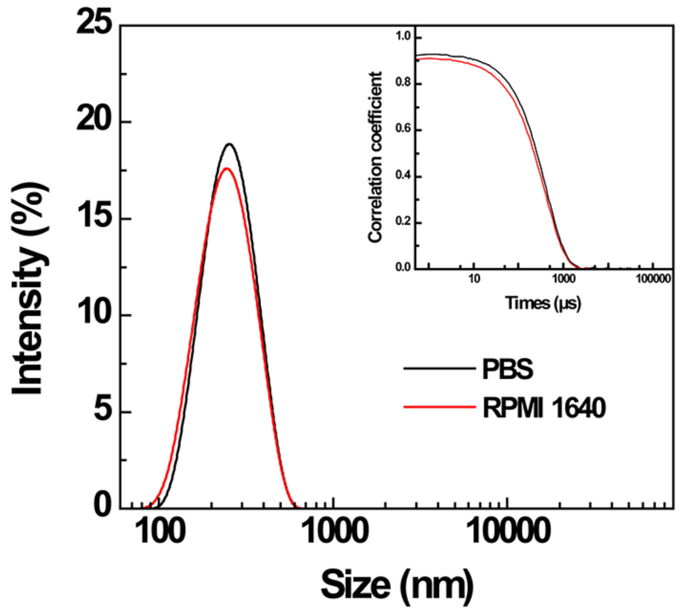

Figure S1. Hydrodynamic size distribution and relative correlation coefficient (inset) of DOX/MnO_2@PVCL NGs in PBS and RPMI 1640 medium.

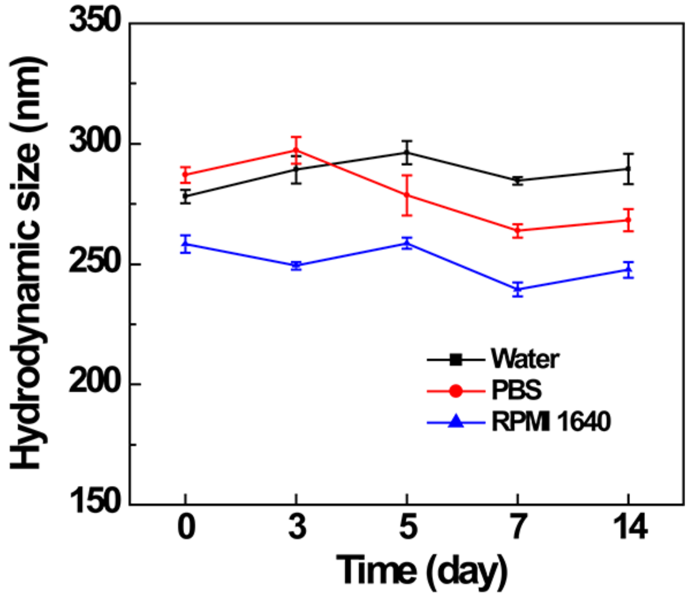

Figure S2. Hydrodynamic size change of DOX/MnO_2@PVCL NGs in water, PBS and RPMI 1640 medium over a period of 14 days (n = 3).

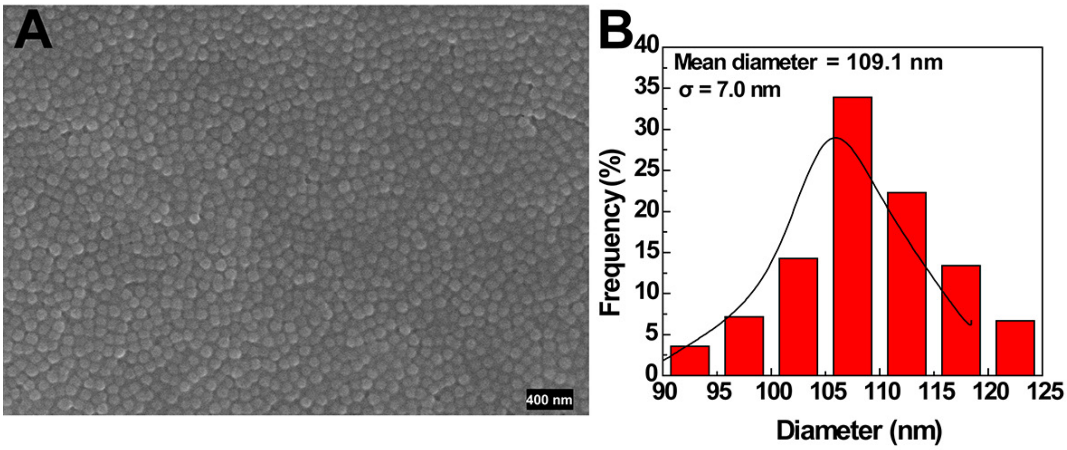

Figure S3. (A) SEM image and (B) size distribution histogram of pristine PVCL NGs.

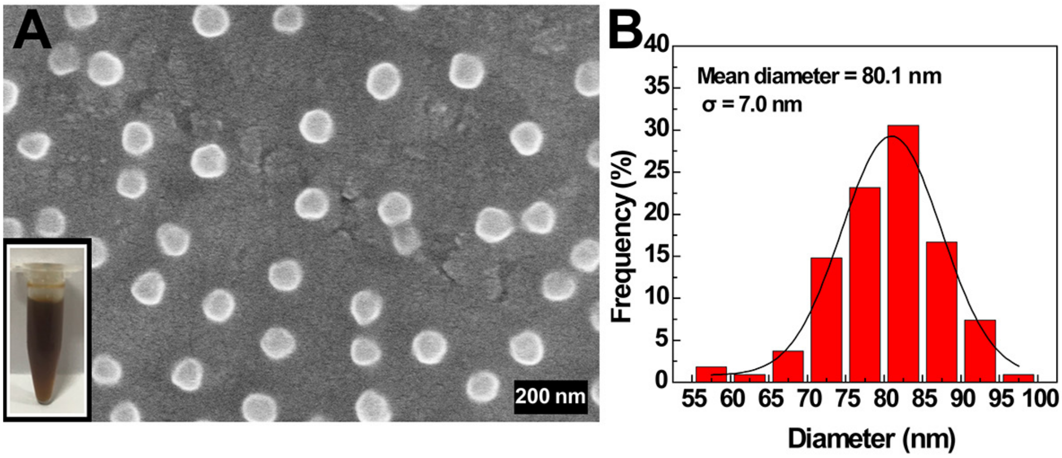

Figure S4. (A) SEM image (solution photograph, inset) and (B) size distribution histogram of MnO_2@PVCL NGs.

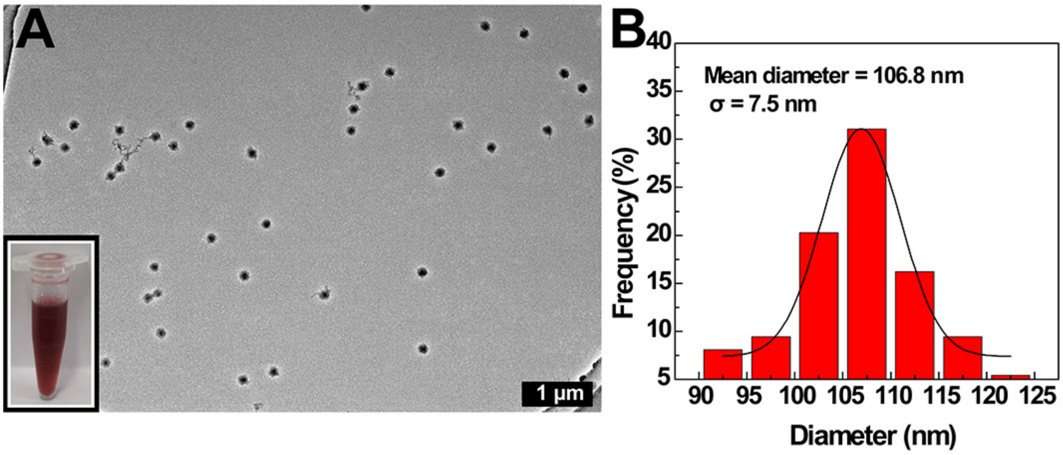

Figure S5. (A) TEM image (solution photograph, inset) and (B) size distribution histogram of DOX/MnO_2@PVCL NGs.

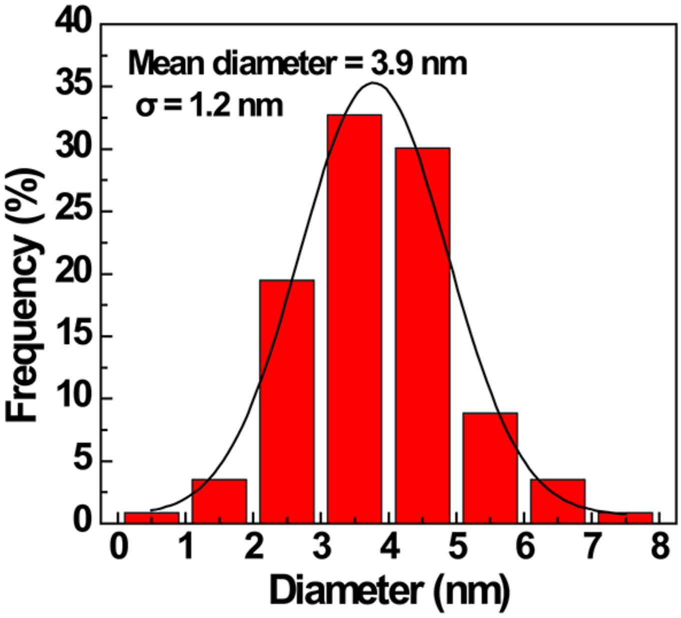

Figure S6. Size distribution histogram of MnO_2 nanoparticles.

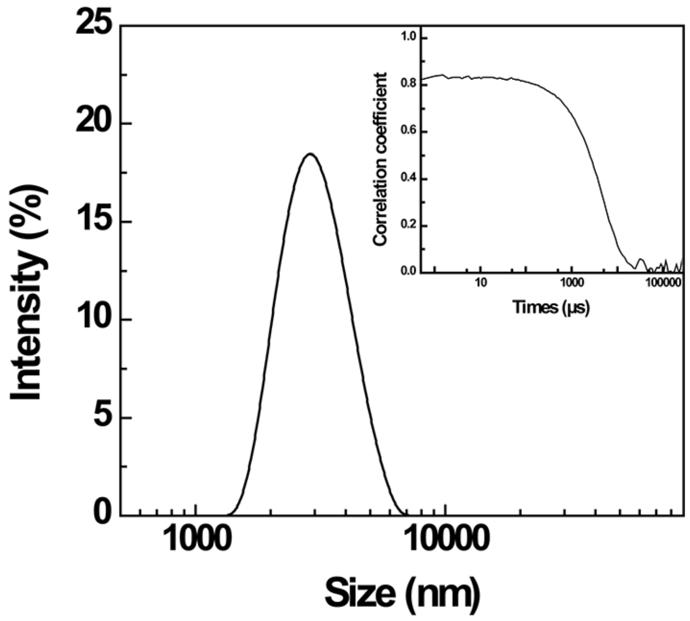

Figure S7. Hydrodynamic size distribution and relative correlation coefficient (inset) of DOX/MnO_2@PVCL NGs in water containing GSH (10 mM).

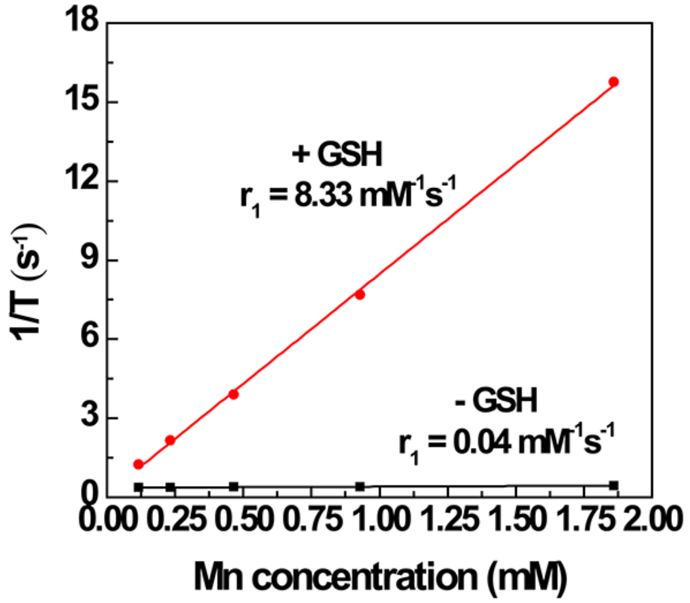

Figure S8. Linear fitting of $1/T_1$ of DOX/MnO_2@PVCL NGs as a function of Mn concentration in the presence and absence of GSH (10 mM).

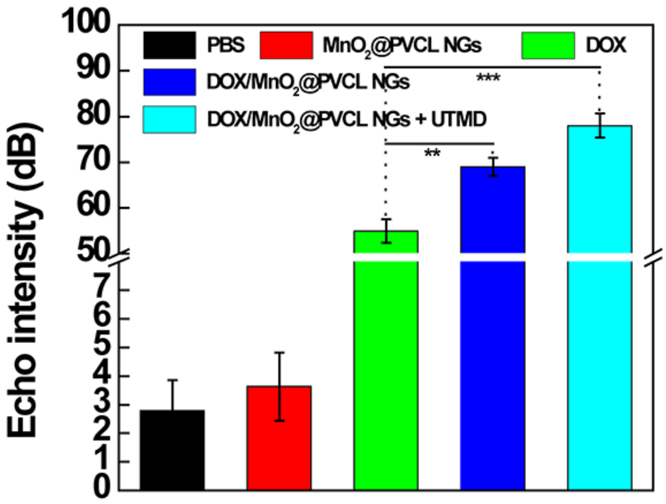

Figure S9. Quantified CUES imaging echo intensity of the tumor site on day 12 after different treatments (n = 3).

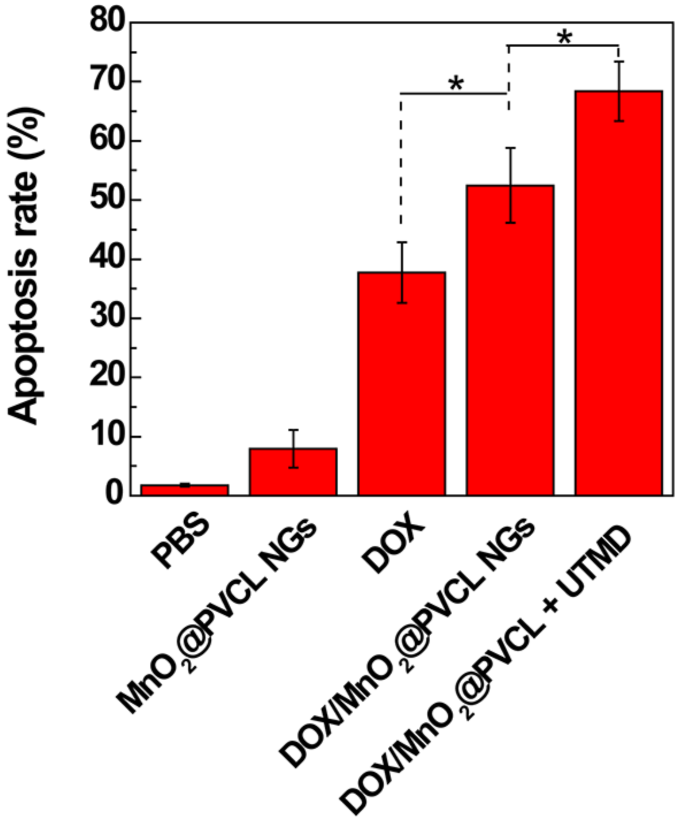

Figure S10. Quantitative analysis of the apoptosis rate of tumor cells after different treatments (n = 3).

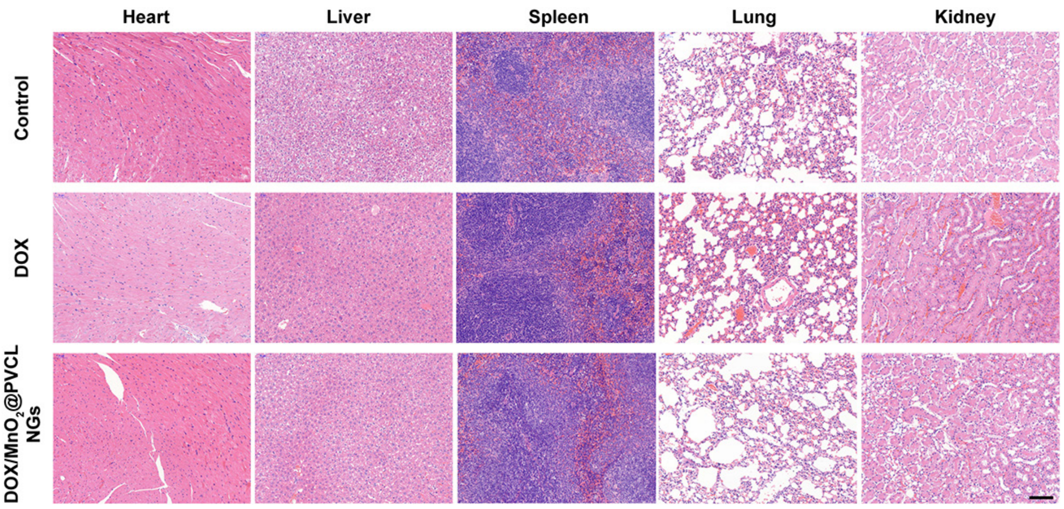

Figure S11. H&E staining of thin sections of the heart, liver, spleen, lung and kidney from B16 tumor-bearing mice treated with free DOX and DOX/MnO_2@PVCL NGs for 12 days. Mice injected with PBS were used as control. Scale bar denotes 100 μm in each panel.

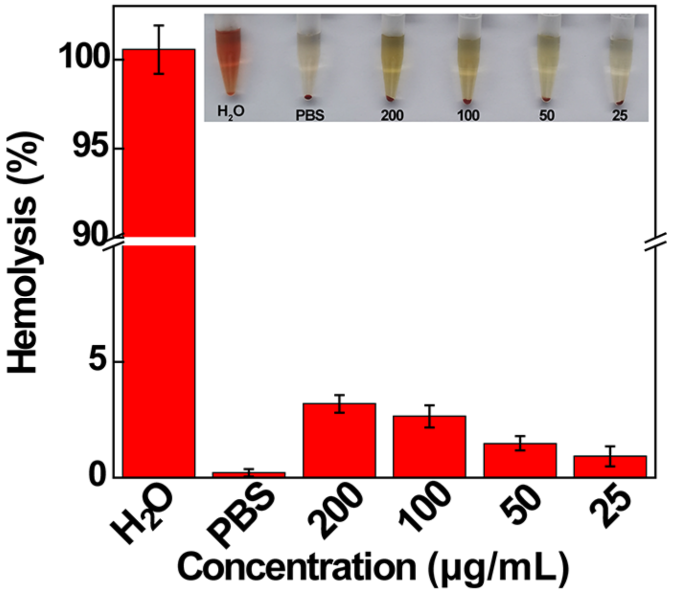

Figure S12. Hemolysis percentages of RBCs after they were treated with the MnO_2@PVCL NGs at different NG concentrations for 2 h (n =3). Inset shows the photograph of RBCs treated with the NGs at the corresponding concentrations after centrifugation. Water and PBS were used as positive and negative controls, respectively.

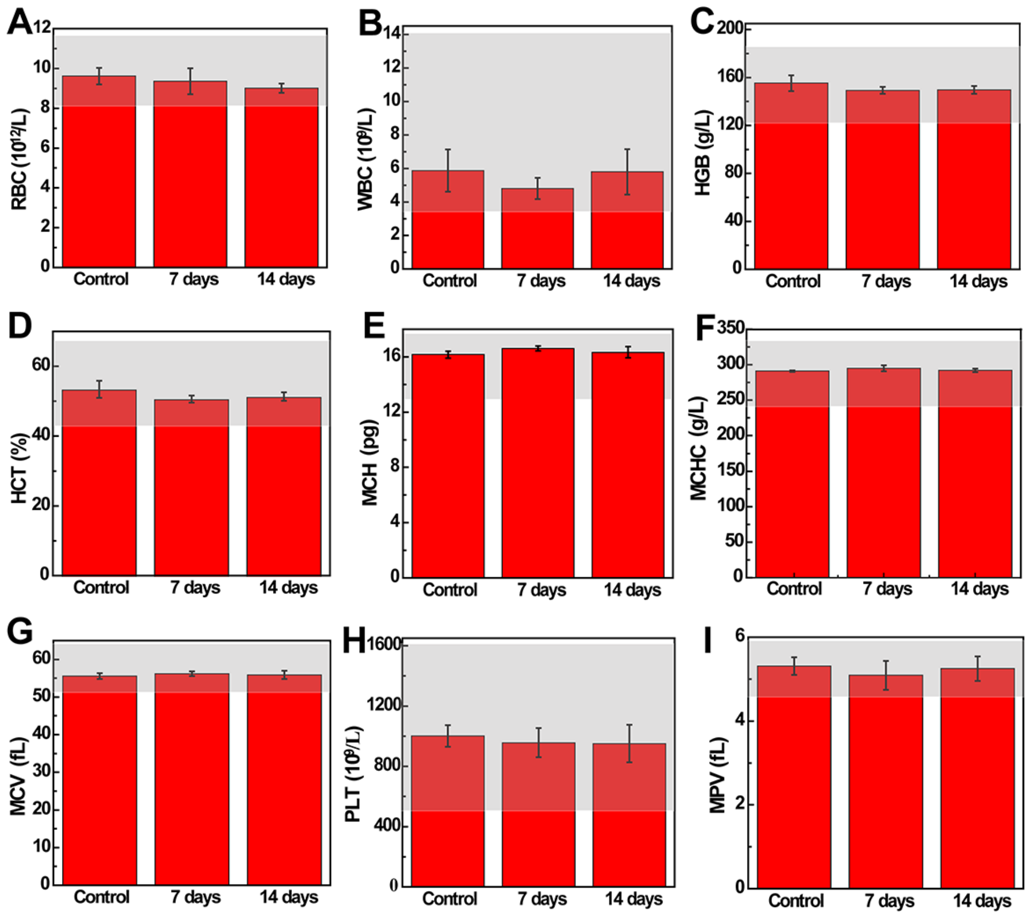

Figure S13. *In vivo* toxicology study. Healthy mice were intravenously injected with PBS as control or DOX/MnO_2@PVCL NGs and were sacrificed at 7 and 14 days post-injection for blood collection and analysis. The blood routines include (A) red blood cells (RBC), (B) white blood cells (WBC), (C) hemoglobin (HGB), (D) hematocrit (HCT), (E) mean corpuscular hemoglobin (MCH), (F) mean corpuscular hemoglobin concentration (MCHC), (G) mean corpuscular volume (MCV), (H) platelet (PLT), and (I) mean platelet volume (MPV). Gray areas indicate the referred normal ranges for healthy mice obtained from Servicebio, Inc. using Hematology Analyzer (Mindray, BC-2800 vet). The data are shown as mean ± SD (n = 3).

References

1. Zhang C, Gau E, Sun W, Zhu J, Schmidt BM, Pich A, et al. Influence of size, crosslinking degree and surface structure of poly(N-vinylcaprolactam)-based microgels on their penetration into multicellular tumor spheroids. Biomater Sci. 2019; 7: 4738-47.

2. Sun W, Thies S, Zhang J, Peng C, Tang G, Shen M, et al. Gadolinium-loaded poly(n-vinylcaprolactam) nanogels: Synthesis, characterization, and application for enhanced tumor mr imaging. ACS Appl Mater Interfaces. 2017; 9: 3411-8.

3. Song M, Liu T, Shi C, Zhang X, Chen X. Bioconjugated manganese dioxide nanoparticles enhance chemotherapy response by priming tumor-associated macrophages toward M1-like phenotype and attenuating tumor hypoxia. ACS Nano. 2016; 10: 633-47.

4. Lin L, Fan Y, Gao F, Jin L, Li D, Sun W, et al. UTMD-promoted co-delivery of gemcitabine and miR-21 inhibitor by dendrimer-entrapped gold nanoparticles for pancreatic cancer therapy. Theranostics. 2018; 8: 1923-39.

5. Ma J, Xing L, Shen M, Li F, Zhu M, Jin L, et al. Ultrasound contrast-enhanced imaging and in vitro antitumor effect of paclitaxel-poly(lactic-co-glycolic acid)-monomethoxypoly (ethylene glycol) nanocapsules with ultrasound-targeted microbubble destruction. Mol Med Rep. 2015; 11: 2413-20.

6. Xing L, Shi Q, Zheng K, Shen M, Ma J, Li F, et al. Ultrasound-mediated microbubble destruction (UMMD) facilitates the delivery of CA19-9 targeted and paclitaxel loaded mPEG-PLGA-PLL nanoparticles in pancreatic cancer. Theranostics. 2016; 6: 1573-87.

7. Cai H, Li K, Li J, Wen S, Chen Q, Shen M, et al. Dendrimer-assisted formation of Fe_3O_4/Au nanocomposite particles for targeted dual mode CT/MR imaging of tumors. Small. 2015; 11: 4584-93.
